# Robust Non-Rigid Point Set Registration Using Student's-t Mixture Model

**DOI:** 10.1371/journal.pone.0091381

**Published:** 2014-03-11

**Authors:** Zhiyong Zhou, Jian Zheng, Yakang Dai, Zhe Zhou, Shi Chen

**Affiliations:** Suzhou Institute of Biomedical Engineering and Technology, Chinese Academy of Sciences, Suzhou, China; Institute of Automation, Chinese Academy of Sciences, China

## Abstract

The Student's-t mixture model, which is heavily tailed and more robust than the Gaussian mixture model, has recently received great attention on image processing. In this paper, we propose a robust non-rigid point set registration algorithm using the Student's-t mixture model. Specifically, first, we consider the alignment of two point sets as a probability density estimation problem and treat one point set as Student's-t mixture model centroids. Then, we fit the Student's-t mixture model centroids to the other point set which is treated as data. Finally, we get the closed-form solutions of registration parameters, leading to a computationally efficient registration algorithm. The proposed algorithm is especially effective for addressing the non-rigid point set registration problem when significant amounts of noise and outliers are present. Moreover, less registration parameters have to be set manually for our algorithm compared to the popular coherent points drift (CPD) algorithm. We have compared our algorithm with other state-of-the-art registration algorithms on both 2D and 3D data with noise and outliers, where our non-rigid registration algorithm showed accurate results and outperformed the other algorithms.

## Introduction

Point set registration is a key component in many tasks such as medical image registration [1], image analysis, computer graphics [2] and shape recognition [3]. Iterative closest point (ICP) algorithm [4], which assigns correspondences with the closest distance criterion, is a popular algorithm for point set registration because of its low computation complexity. However, ICP requires the initial positions of the two point sets to be adequately close. Gold et al [5] proposed the robust point matching (RPM) algorithm that performs soft-assignment of correspondences and transformation alternately. Chui et al [6] pointed out that the processing of alternating soft-assignment of correspondences and transformation in the RPM algorithm is equivalent to the Expectation Maximization (EM) algorithm with Gaussian mixture model (GMM), where one point set is considered as GMM centroids and the other one is considered as data. Then Chui et al [7] re-parameterized the deformation field using Thin-Plate-Spline (TPS) and resulted in the TPS-RPM algorithm. Revow et al [8] represented the contour-like point sets using splines and modeled them by the probabilistic GMM formulation, where GMM centroids were uniformly positioned along the contour. This algorithm allows non-rigid transformation between point sets. Tsin and Kanade [9] proposed a correlation-based point set registration approach, which was later improved by Jian and Vemuri [10] (TPS-L2). This approach treats the non-rigid point set registration as the minimization of the L2 distance between two Gaussian mixture models, and then parameterizes the point sets using explicit TPS parameterizations.

Recently, GMM is widely used to model the non-rigid point set registration as it is a natural and simple way to represent the given point sets. However, most of the GMM-based non-rigid registration algorithms ignore significant effect of the outliers and noise. Myronenko and Song [11] introduced the coherent point drift (CPD) algorithm, which enforces the points to drift coherently by regularizing the deformation field based on the motion coherence theory (MCT) [12], [13]. The CPD algorithm models the noise and outliers using a uniform distribution and uses a manually defined parameter *ω* to denote the amount of noise and outliers. However, the non-rigid registration is significantly sensitive to *ω* and it is an arduous task to obtain the optimal value of *ω*. Wang Peng [14] introduced a hybrid optimization method to estimate the parameter *ω*. But, the hybrid optimization algorithm slows the non-rigid registration. Christopher et al [15] introduced a Student's-t mixture model (SMM) that is a finite mixture model based on the Student's-t distribution. The SMM is heavily tailed and more robust against noise and outliers than GMM. Gerogiannis et al [16], [17] proposed a SMM-based rigid registration algorithm. However, their algorithms (Christopher et al [15] and Gerogiannis et al [16], [17]) can only obtain parameters such as data mean, covariance matrix and mixing proportion, which limits the algorithm to rigid registration.

It's indicated that, theoretically, GMM is a special case of the SMM [15]. Peel and McLachlan [18] modeled point sets with outliers and noise for cluster analysis by using SMM. They later treated the SMM as a weighted GMM integral form in order to obtain closed-form solutions. But unfortunately, they did not extend their approach for non-rigid point set registration. To date, it is still a challenge to obtain closed-form solutions for the non-rigid point set registration by using the SMM [18]–[20]. In this paper, we propose a novel SMM-based non-rigid point set registration algorithm. Specifically, we formulate most of the registration parameters, such as the deformation field, the equal isotropic covariance, and the degree of freedom of the Student's-t distribution, and find their closed-form solutions using the EM algorithm. The proposed algorithm is computationally efficient and more robust against significant amount of outliers and noise than existing algorithms.

The rest of this paper is organized as follows. We introduce the main idea of the Student's-t mixture model for non-rigid point set registration and details of the proposed method in section 2. Section 3 demonstrates experimental results on 2D contour-like point sets, 3D surface-like point sets and 3D cloud-like point sets. Finally, we present discussion and conclusions in section 4.

## Methods

### 1 Student's-t mixture model

The first point set ***X*** *_N_*_×*D*_ = (***x***_1_,…***x*** *_N_*)*^T^* is the *D*-dimension data considered as a data point set. The other point set ***Y*** *_M_*_×*D*_ = (***y***_1_,…***y*** *_M_*)*^T^* is the *D*-dimension SMM centroid set. *N* and *M* are the numbers of points in ***X*** and ***Y***, respectively. The GMM probability density function is defined as
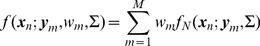
(1)where 
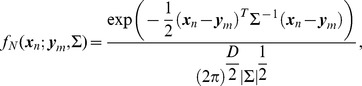
 Σ is a covariance matrix, and *w_m_* is a prior probability for ***y*** *_m_*. *w_m_* satisfies the following constraint



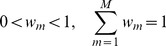
(2)If the point sets contain outliers or noise, we model both the data set and the outliers (or noise) set using two Gaussian mixture models as follows
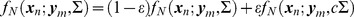
(3)where *ε* is a small value and *c* is a large value. 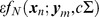 represents the outliers and noise in point sets. In brief, we model the outliers and noise using the GMM with a different covariance matrix.

**Figure 1:**
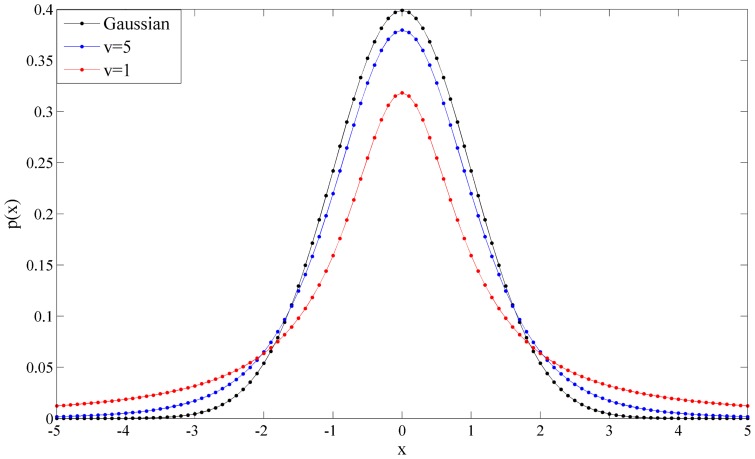
A univariate Student's-t distribution (*μ* = 0, *σ* = 1) for various degrees of freedom. The Student's-t distribution corresponds to a Gaussian distribution when *γ*→∞.

A random variable *u* following the Gamma distribution is defined if there are outliers and noise in point sets. Specifically, we assume *u*∼*f*_Γ_(*α*, *β*), where *α* and *β* are set as *α* = *β* = *γ*/2. Combining the definition to equation (3), equation (1) can be rewritten as
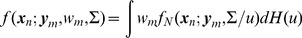
(4)where *H*(*u*) is *χ* ^2^ distribution. The probability density function of the Student's-distribution mixture model is
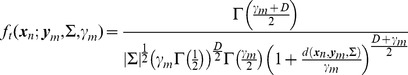
(5)where Γ(•) is a Gamma function, γm is the degree of freedom of Student's-distribution for ym, which can change its distribution model to fit the data points, and d(x,y,Σ) = (x-y)TΣ−1(x-y) is a Mahalanobis square distance. We assume Σ = σ^2^I, so as to simplify the Student's-t distribution for all SMM components. Then the SMM takes the form



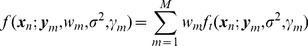
(6)
Figure 1 shows the probability density of a univariate Student's t-distribution for various degrees of freedom.

Mathematically, the Student's-t distribution is equivalent to the Gaussian distribution when *γ*→∞, and it is also equivalent to standard Cauchy distribution when *γ* = 1 [21]. The SMM is more robust than the GMM due to its heavy tail for significant amount of outliers and noise. According to equation(4), the SMM can be represented as a weighted Gaussian mixture model with a Gamma distribution and closed-form solutions can then be obtained by using the EM algorithm.

### 2 Likelihood function for Student's-t mixture model

We introduce the completion data *X_c_* in EM framework to obtain the closed-form solutions. *X_c_* is defined as *X_c_* = {***X***, *z*_1_, …, *z_N_*_,_ *u*_1_, …, *u_N_*}, where *z_mn_* = (*z_n_*)*_m_*. *z_mn_* = 1 if ***x*** *_n_* is corresponding to ***y*** *_m_*, otherwise *z_mn_* = 0. *u_i_* (*i* = 1,2,…,*N*) is a random variable following Gamma distribution to scale the Gaussian distribution. As denoted in [22], we have
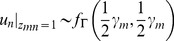
(7)




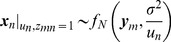
(8)


The likelihood function *L* by parameterization is denoted as follows
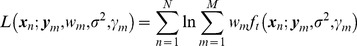
(9)


The parameter set for non-rigid point set registration is defined as Ψ =  (*w*_1_, …, *w_M_*, *γ*_1_,…, *γ_M_*, ***y***_1_,…, ***y*** *_M_*, *σ* ^2^). We separate parameters from each other in the SMM likelihood function by
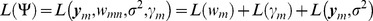
(10)and get the following formulas by combining equations(5), (7) and (8)



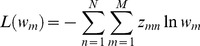
(11)

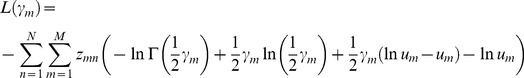
(12)




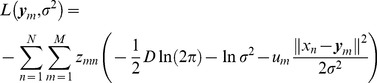
(13)


### 3 Estimation of parameters

We maximize the conditional expectation of parameters in the EM algorithm for estimation of parameters of non-rigid registration. The conditional expectation in the *k*+1 iteration is given by
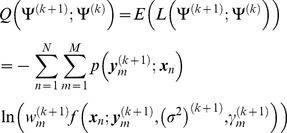
(14)where 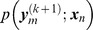 is a posterior probability density function that is denoted as



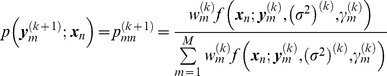
(15)By maximizing equation (14), we can obtain the update equations of 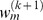,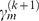,
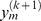
and 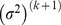. In equation (14), 
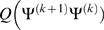
 is a function with respect to 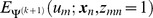
and 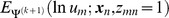. So we have to calculate 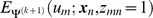 and 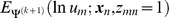 for the maximization of
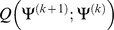
. Because *u* is the conjugate prior distribution with respect to *f*_Γ_, we can formulate 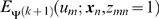 as follows
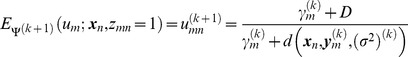
(16)


Using equation (16), 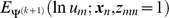 is denoted as
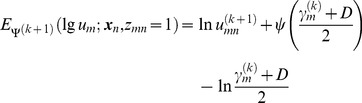
(17)where *ψ*(•) is a Digamma function and 
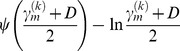
 is a correction for ln*u_m_*.

We separate *w*, *γ*, and *σ* ^2^ from each other for equation (14), which can then be rewritten as
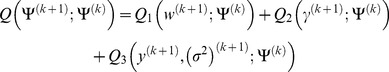
(18)where *Q*_1_, *Q*_2_, and *Q*_3_ are respectively



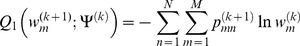
(19)

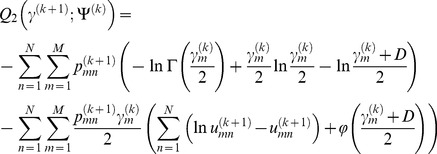
(20)




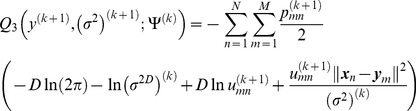
(21)


In equation (19), the update equation of 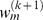 is
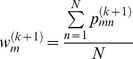
(22)


In equation (20), 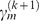 is a solution to
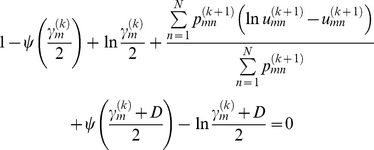
(23)


Implementation details of equation (21) and how to force the SMM centroids move coherently as a group are described in the following subsection.

### 4 Non-rigid registration with SMM

The deformation field ***T*** of the non-rigid point set registration is assumed as ***T***(***Y***+***v***) = ***Y***+***v***(***Y***), where ***v*** is the deformation vector set. The regularization *φ*(***Y***) regularizes the norm of ***v***, leading to a smoothness deformation field that enforces the SMM centroids to move coherently as a group to preserve the topological structure of the point set [23], [24]. Adding the regularization *φ*(***Y***) to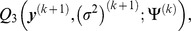 equation (21) can be rewritten as
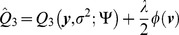
(24)where *λ* is the weight of the regularization representing the trade-off between the goodness of non-rigid registration and regularization. After some manipulations similar to [11], 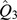
can be rewritten as
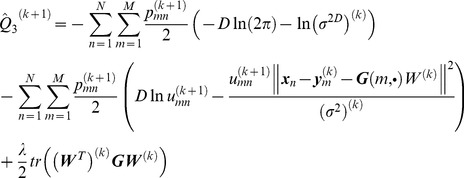
(25)where GM×M is a Gaussian kernel matrix with elements 
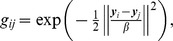
 which reduces the oscillating energy at high frequency [24]. G(m,•) is a column vector in the kernel GM×M. The parameter β defines the width of smoothing Gaussian filter in [25]. WM×D is the weight matrix of GM×M. Using 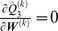, *W* ^(*k*)^ can be given by
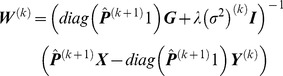
(26)where 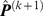 is a M×N matrix with elements 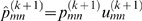, representing the posterior probability density correction by 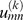. 1 is a column vector of all ones. I is an identity matrix. diag(⋅) represents a diagonal matrix. Using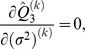 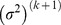 is denoted as



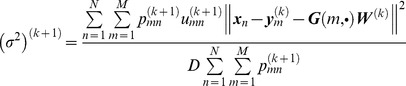
(27)Theoretically, *x_n_* is considered as an observation in Bayesian approach. The discrete latent variable *z_mn_* specifies which component of the Student's-t mixture model generates *x_n_*, and the continuous latent variable *u_mn_* specifying the scaling for the corresponding equivalent Gaussian [15]. Consequently, ***W*** and *σ* ^2^ in our algorithm respectively correspond to the ones of the CPD algorithm if *z_mn_* = 1 and *u_mn_* = 1.

Hence, the main steps in this proposed algorithm are summarized as follows.

**Step 1:**

Initialize the parameter set Ψ =  (*γ_m_*, *w_m_*, *σ* ^2^,***v***), *β*, *λ* and the convergence threshold *τ*. The initial values of 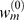
and 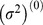
are 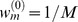 and 
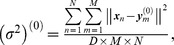
 respectively.

**Step 2:**

Construct ***G***: 
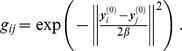


**Step 3:**

Calculate the probability density for point sets using equation (5).

**Step 3: E-Step.**

Calculate posterior probability density 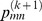 using equation (15) and 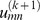 using equation (16), respectively.

**Step 4: M-Step.**

Estimate 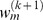 using equation (22), 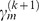 using equation (23), ***W*** ^(*k*+1)^ using equation (26), and (*σ* ^2^)^(*k*+1)^ using equation (27), respectively.

**Step 5:**

Calculate the new positions of the SMM centroids using ***Y*** ^(*k*+1)^ = ***Y*** ^(*k*)^+***G*** ^(*k*+1)^ ***W*** ^(*k*+1)^.

**Step 6:**

Return to step 3 if the registration error *e*>*τ*.

## Results

To compare our SMM-based algorithm with CPD and TPS-RPM for non-rigid point set registration, we performed experiments on 2D contour-like point sets with outliers and 3D surface point sets with noise.

### 1 Qualitative evaluation

Here we present qualitative comparisons of the non-rigid registration algorithms. The experiments were conducted on 2D contour-like point sets, which were from the boundary of cerebrospinal fluid (CSF) segmented from CT images.

**Figure 2:**
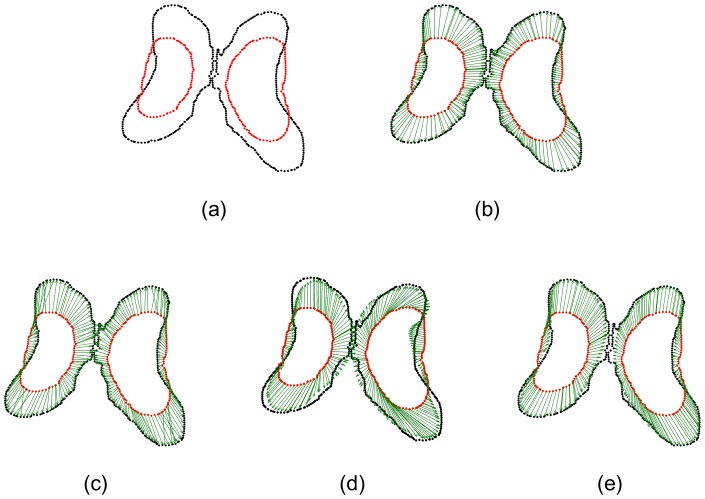
Registration of 2D CSF boundary points without outliers or noise. The 2D CSF points are segmented on the images from http://www.insight-journal.org/rire/download.php. (a) Ideal CSF boundary points, the black point set contains 488 points and the red one contains 398 points. (b) The registration of our SMM-based algorithm (*β* = 2.2, *λ* = 2), (c) CPD, (d) the Jian and Verimu's algorithm [10] (TPS-L2), (e) TPS-RPM. The green vectors denote the deformation vectors produced by non-rigid registration algorithms.

In the first experiment, we segmented CSFs very carefully and got a quite ideal segmentation result. The corresponding point sets are shown in Figure 2(a). The black point set had 488 points and the red one had 398 points. The two point sets were considered as the ideal boundary of CSF without any outliers or noise. However, the boundary in the central region (as indicated by the arrow in Figure 2(a)) was tortuous, which caused the CPD algorithm to fail to align correspondences between two point sets. Consequently, the deformation field obtained by the CPD algorithm was aliasing and the topological structure was changed. In Figure 2(d), several correspondences in the margin are improperly aligned by using TPS-L2. Most of red points were aligned accurately by the TPS-RPM algorithm due to its TPS regularization. But because of its explicit TPS regularization, the boundary of the central region is too smooth to fit data points. Our SMM-based algorithm aligned the central points more accurately than TPS-RPM and the deformation field produced by our algorithm was smoother than the one produced by the CPD algorithm. We set *β* = 2.2 and *λ* = 2 in the qualitative experiments. The deformation vectors (as indicated by the green vectors) are also displayed in Figure 2(b) ∼ Figure 2(e).

**Figure 3:**
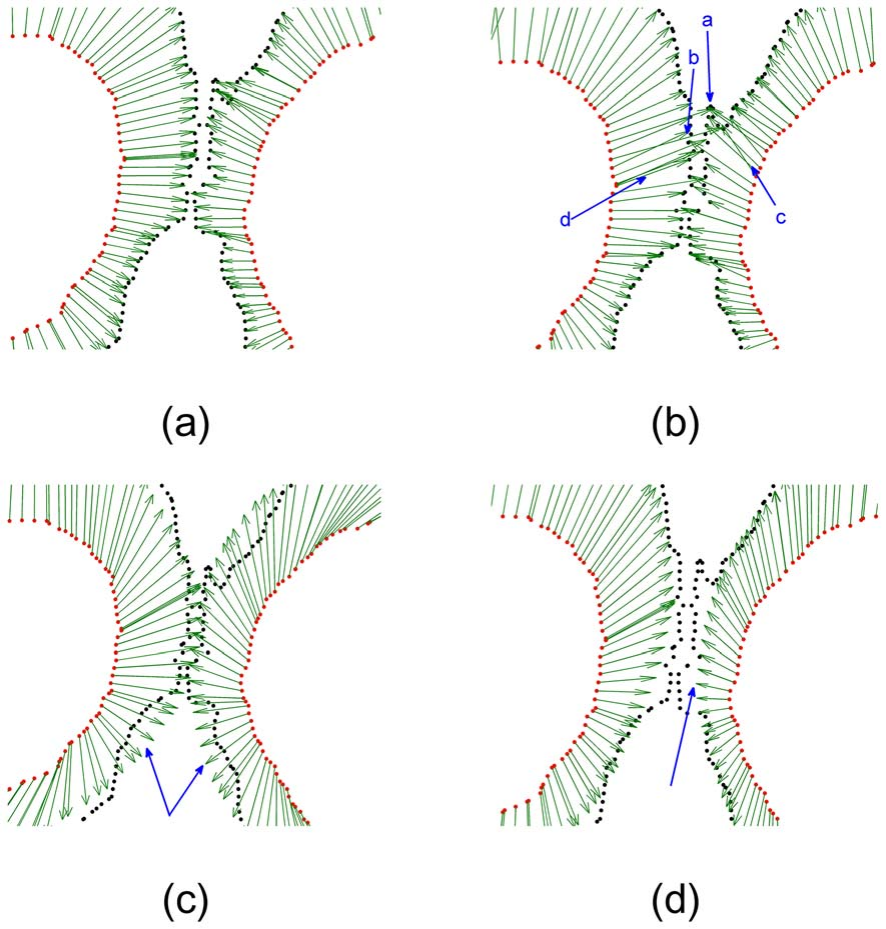
Deformation vectors of central points. (a) Our algorithm, (b) CPD, (c) TPS-L2, (d) TPS-RPM. The deformation vectors produced by our algorithm are more regularized than the ones produced by CPD, and the registration result of our algorithm is more accurate than TPS-L2 and TPS-RPM. Some deformation vectors produced by CPD were crossed, which broke the topological structure of the point sets. The aligned point set was too smooth to fit the CSF boundary points by using TPS-RPM.

The deformation vectors produced by the four algorithms are zoomed in for the central points in Figure 3. All points were aligned properly by our algorithm (see Figure 3(a)). However, some points were aligned improperly (as indicated by the arrows a and b in Figure 3(b)) by the CPD algorithm, and several vectors were crossed (as indicated by the arrows c and d in Figure 3(b)), which broke the topological structure of the aligned point set. The deformation vectors produced by TPS-L2 are more regularized than the ones produced by our SMM-based algorithm and the CPD algorithm. However, the TPS-L2 algorithm failed to align a few correspondences in the central region (as indicated by the arrow in Figure 3(c)). Similar to TPS-L2, the deformation vectors produced by the TPS-RMP algorithm are regularized, but TPS-RMP also failed to align the central points (as indicated by the arrow in Figure 3(d)). Obviously, our SMM-based registration algorithm is the most accurate one of the four algorithms.

**Figure 4:**
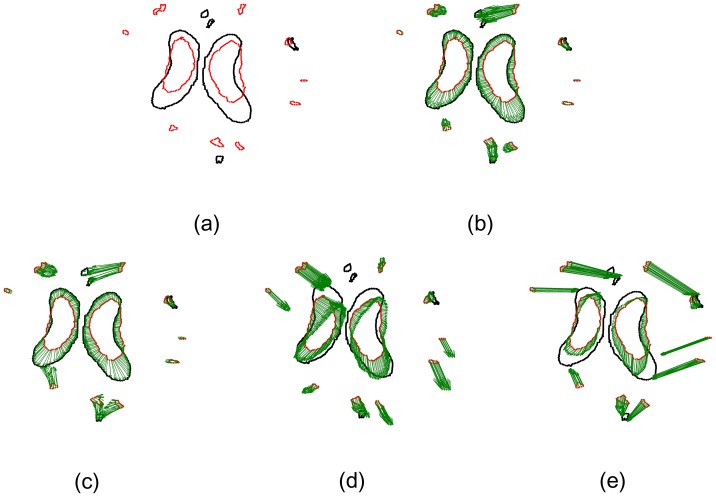
Registration of CSF boundary point sets with outliers. (a) CSF boundary point sets with outliers, 184 red outliers clustered into 11 outlier sets, and 54 black points clustered into 4 outlier sets. (b) Our algorithm, (c) CPD, (d) Jian and Verimu's algorithm [7] (TPS-L2), (e) TPS-RPM. One outlier set was aligned to the CSF boundary points by CPD, and two outlier sets are aligned to the boundary points by TPS-L2 and TPS-RPM.

Generally, segmentation results are sensitive to image quality and the selection of parameters of segmentation algorithms. Consequently, we may not segment targets very accurately and obtain ideal point sets without outliers and noise. In the second experiment, the comparison of the registration algorithms was performed on the point sets of CSF boundary containing outliers (see Figure 4(a)). There were 184 outliers clustered into 11 outlier sets in the red point set and 54 outliers clustered into 4 outlier sets in the black point set. The registration result by our algorithm is shown in Figure 4(b). Figure 4(c) (the registration result by the CPD algorithm) shows that one outlier set (as indicated by the arrow) are shifted towards the CSF boundary, which implies the CPD algorithm cannot align the correspondences between the point sets when the counter-like point sets contain outliers. Figure 4(d) shows the registration result (which is the worst) by the TPS-L2 algorithm, indicating that outliers could seriously affect the TPS-L2 algorithm. Similar to Figure 4(d), Figure 4(e) also shows a bad registration result by the TPS-RPM algorithm, indicating that the TPS-RPM algorithm failed to parameterize the deformation field of CSF boundary exactly.

**Figure 5:**
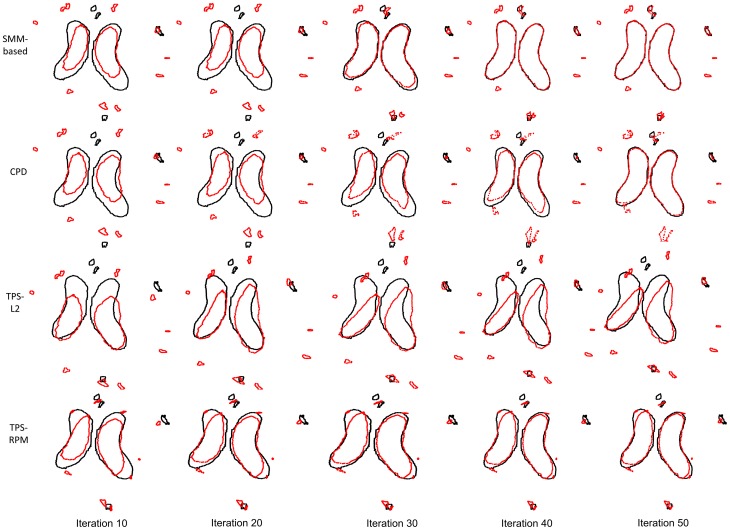
Iterations of the four non-rigid registration algorithms. The convergence of our algorithm takes 40 iterations, while the CPD algorithm takes about 50 iterations and the TPS-L2 algorithm and the TPS-RPM algorithm takes more than 50 iterations.

Figure 5 shows the iterations of the four non-rigid registration algorithms. The first, second, third and fourth rows demonstrate the iterations of our SMM-based algorithm, the CPD algorithm, the TPS-L2 algorithm and the TPS-RPM algorithm, respectively. The degree of the freedom *γ* can change the Student's-t distribution model to fit the data points [26], making the convergence of our algorithm only took 40 iterations, less than the CPD algorithm, and significantly less than the TPS-L2 algorithm and TPS-RPM algorithm.

### 2 Quantitative evaluation

#### 2.1 3D face point set registration

In this experiment, we demonstrate the quantitative performance of the algorithms on 3D face data with 392 points (which are from http://www.csee.ogi.edu/myron/matlab/cpd/). We define the registration error as
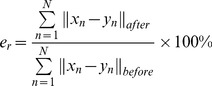
(28)where 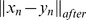 denotes the Euclidean distance of the correspondences in ***X*** and ***Y*** after registration, 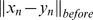 denotes the Euclidean distance of the correspondences before registration, and *N* is the amount of points in each point set.

**Figure 6:**
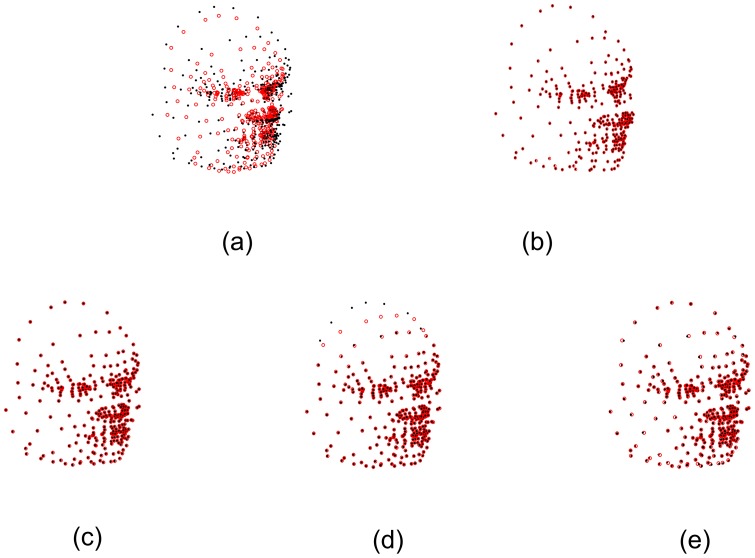
Registration of 3D face data sets with 392 data points. (a) 3D face data set, (b) our algorithm (*β* = 2.2, *λ* = 3, *e_r_* = 1.27%), (c) CPD(*e_r_* = 2.53%), (d) TPS-L2(*e_r_* = 3.11%), (d) TPS-RPM(*e_r_* = 3.54%).

Figure 6 shows the non-rigid registration results produced by our SMM-based algorithm, CPD, TPS-L2 and TPS-RPM on the 3D face data, respectively. In the experiment, we set *β* = 2.2 and *λ* = 3, respectively. The registration error is 1.27% by using our SMM-based algorithm, and the errors are 2.53%, 3.11% and 3.54% by using CPD, TPS-L2 and TPS-RPM respectively.

**Figure 7:**
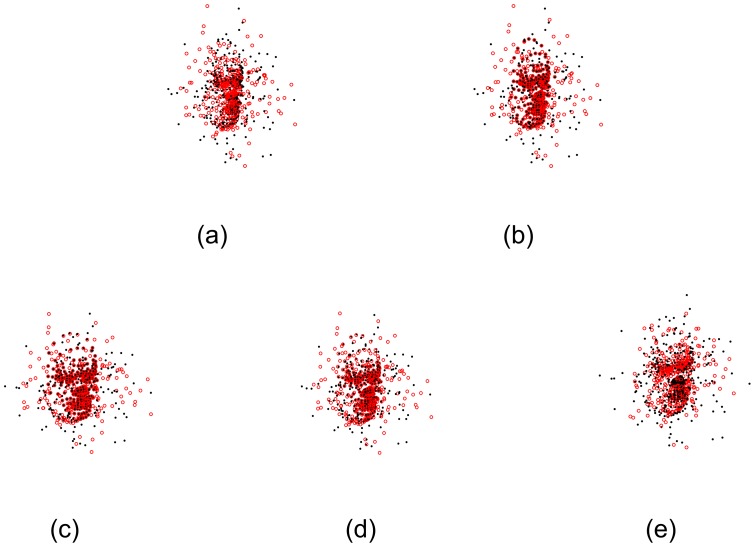
Registration results on 3D face data containing 40% artificially added Gaussian noise with *ó* = 5. (a) 3D face data sets with Gaussian noise (*σ* = 5), (b) our SMM-based algorithm(*e_r_* = 3.36%), (c) CPD(*e_r_* = 10.7%), (d) TPS-L2(*e_r_* = 15.4%), (e) TPS-RPM(*e_r_* = 21.1%).

**Figure 8:**
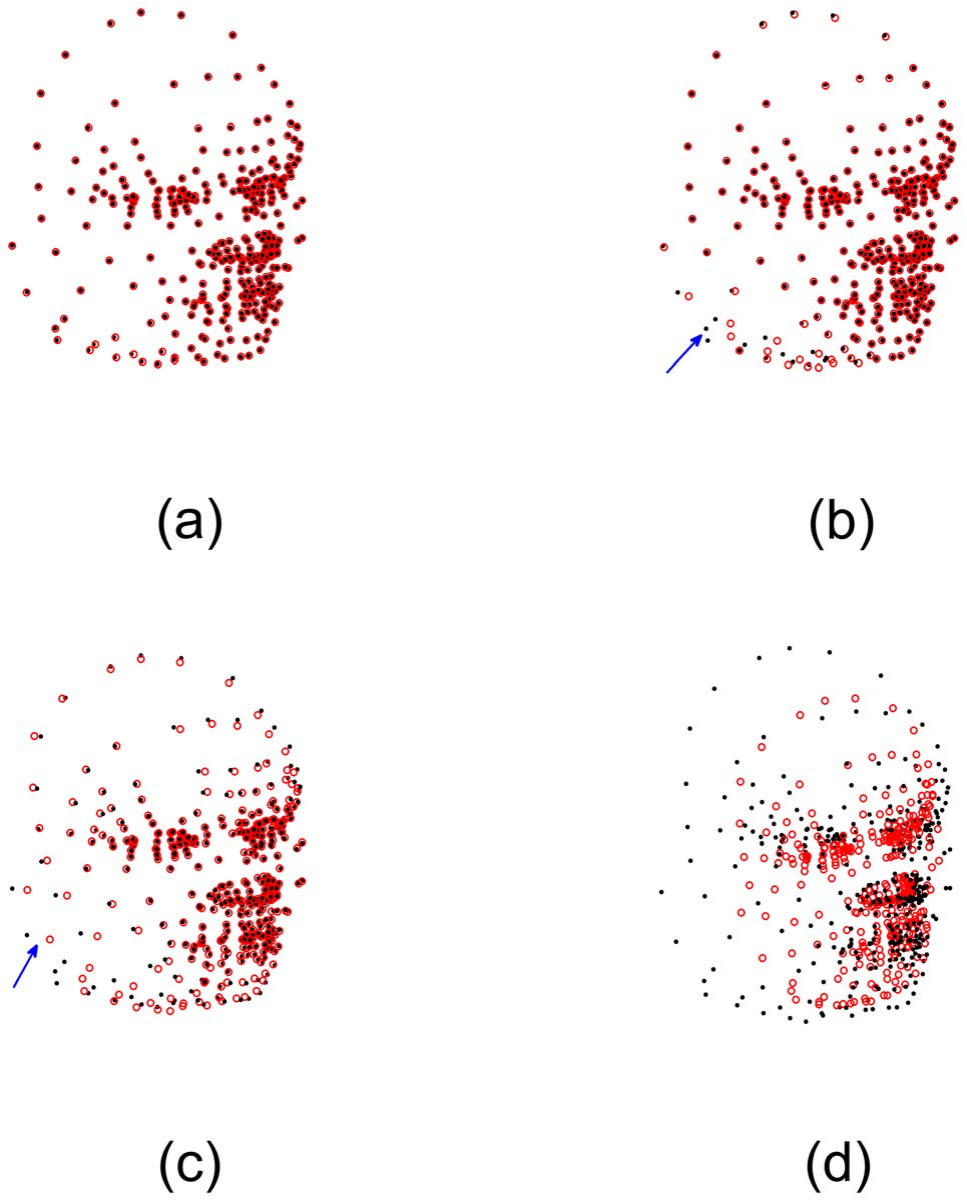
Registration results on 3D face data without the display of artificially added noise. (a) Our SMM-based algorithm (b) CPD, (c) TPS-L2, (d) TPS-RPM. The correspondences are aligned accurately by our algorithm, which demonstrates that our SMM-based algorithm is robust against the significant amount of noise. The CPD algorithm and the TPS-L2 algorithm failed to align some correspondences in the margin, and the TPS-RPM algorithm absolutely failed to align the marginal face points.

Figure 7 shows the registration results on the 3D face data containing 40% artificially added Gaussian noise with *σ* = 5. It is challenging for non-rigid registration algorithms to align 3D points with such noise. Figure 8 shows the registration results on the 3D face data, hiding the artificially added noise. Figure 8(a) demonstrates that our SMM-based algorithm can align the correspondences precisely. Figure 8(b) and Figure 8(c) show that CPD and TPS-L2 are able to align the point sets accurately except few points in the margin (as indicated by the arrow in Figure 8(b) and Figure 8(c)). Figure 8(d) shows a registration with serious error by TPS-RPM, indicating a failure of TPS-RPM for handling the marginal points with so much noise. In Figure 7 and Figure 8, the registration error is 3.36% by using our algorithm, and the errors are 10.7%, 15.4% and 21.1% by using CPD, TPS-L2 and TPS-RPM respectively.

**Figure 9:**
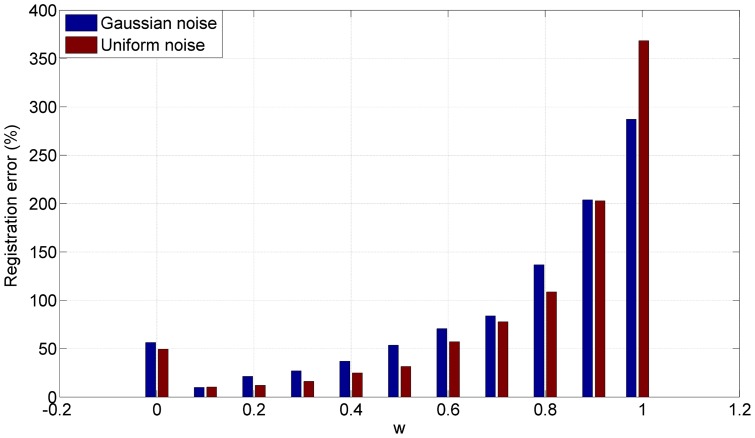
Impacts of *ω* on registration of CPD. The registration results of CPD are sensitive to the parameter *ω*.

**Figure 10:**
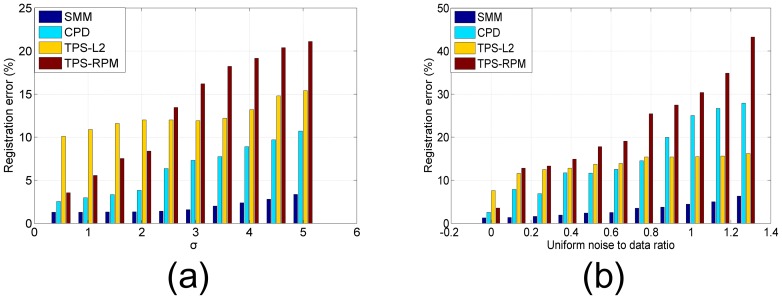
Registration errors on the face data with noise. (a) Registration errors on face data with Gaussian noise, (b) registration errors on face data with uniform noise. Our algorithm is robust against uniform noise and Gaussian noise, and outperformed CPD and TPS-RPM.

A series of experiments demonstrate that the registration of the CPD algorithm is sensitive to the parameter *ω* which indicates the amount of noise in the point sets. However, this parameter had to be set manually. We tested the impacts of *ω* on 3D face data with 40% Gaussian noise and 40% uniform noise, respectively. Figure 9 shows the great impacts of *ω* on registration. In practice, the relationship between the optimal value of *ω* and the amount of noise is nonlinear. It's thus a challenge to get an optimal value of *ω*. Figure 10 shows the registration errors of the four algorithms on the 3D face point sets with Gaussian noise and uniform noise, respectively. The results demonstrate that our algorithm is more accurate and robust than the other algorithms, especially in presence of significant amount of noise.

#### 2.2 3D lung point set registration

**Figure 11:**
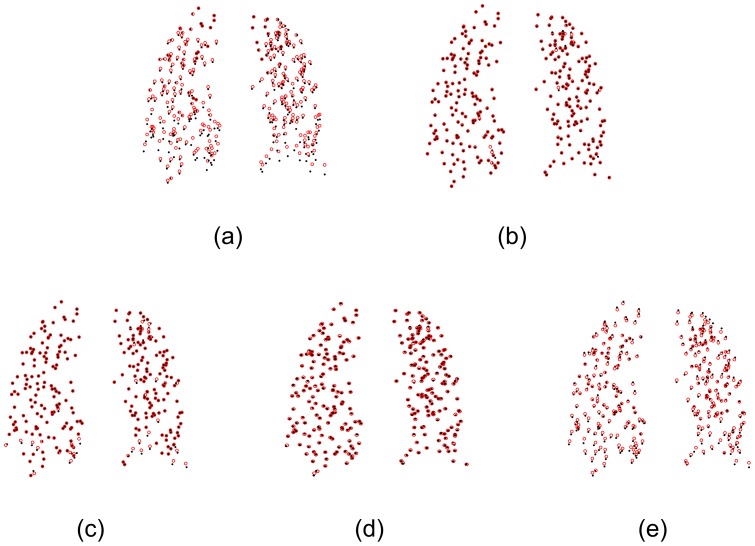
3D lung point set registration on a subject. (a) 3D lung point sets before registration, (b) SMM-based algorithm, (c) CPD, (d) TPS-L2, (e) TPS-RPM. Our algorithm performs the best.

**Figure 12:**
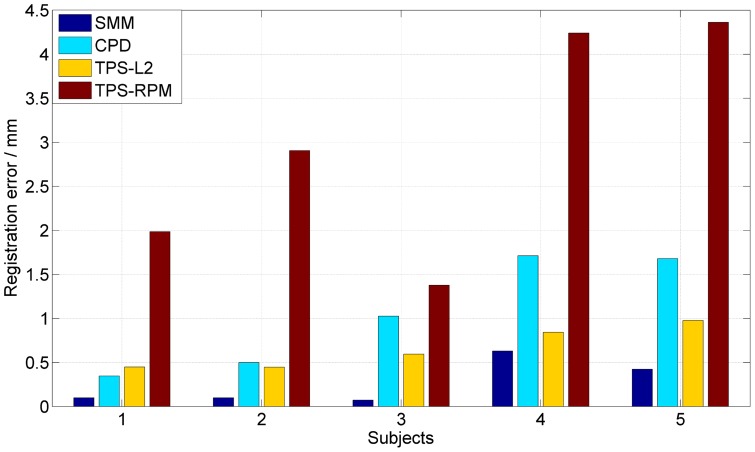
Performance of the four non-rigid registration algorithms on 5 subjects. Our algorithm is the most accurate one of the all.

In this experiment, we demonstrate the quantitative performance of the four algorithms on 5 different 3D lung datasets from 5 different subjects (which are from http://www.dir-lab.com/index.html). Each dataset has a pair of 3D lung point sets, one set was extracted from the maximum inhalation phase image and the other set was extracted from the maximum exhalation phase image. Each 3D lung point set has 300 cloud-like points, which were selected by experts to make the two point sets correspond to each other. It is a herculean task for non-rigid registration algorithms to match cloud-like point sets accurately due to lack of the topological structure or the geometry structure in such sets. For the above reason, we did not add any noise or outliers in these cloud-like point sets. The average registration errors are 0.26 mm, 1.05 mm, 0.66 mm and 2.97 mm by using our SMM-based algorithm, CPD, TPS-L2 and TPS-RPM respectively. Figure 11 shows the non-rigid registration results produced by our SMM-based algorithm and the other three algorithms. Figure 11(b) shows that our algorithm aligned all correspondences accurately. Figure 11(c) and Figure 11(d) show that CPD and TPS-L2 are able to align most correspondences except few points in the bottom of the lung. Figure 11(e) shows a bad registration result by using TPS-RPM, indicating a failure of TPS-RPM for handling these cloud-like points. Figure 12 shows the registration errors of the four algorithms on the five subjects.

## Discussion

The Student's-t mixture model is heavily tailed and more robust than the Gaussian model, and has recently received great attention on image processing. We have proposed a probabilistic method for non-rigid point set registration based on the Student's-t mixture model. We considered the alignment of two point sets as probability density estimation, where one point set was represented as the Students'-t mixture model centroids, and the other one was represented as the data points. We iteratively aligned the SMM centroids by the EM algorithm and estimated the posterior probabilities of centroids, which provided the correspondence probability. Finally we estimated all registration parameters and obtained closed-form solutions. It's worth noting that, in the Student's-t mixture model, we formulate all registration parameters of each point using the expectation maximization (EM) algorithm, making the coherent point drift algorithm modeled by the Gaussian mixture model be a special case of our algorithm. Our algorithm is superior to the other algorithms for addressing the non-rigid point set registration problem, especially when significant amounts of noise and outliers are present.

Our contribution includes the following aspects. Firstly, we modeled the non-rigid point set registration using the Student's-t mixture model (SMM). Our SMM-based algorithm is more robust than CPD, TPS-L2 and TPS-RPM when the point sets contain significant amounts of noise and outliers. Secondly, we formulated all registration parameters of each point using the EM algorithm, making the CPD algorithm to be a special case of our algorithm. Thirdly, we estimated the prior probability *w_m_* in the EM algorithm while it was assumed as a constant in the CPD algorithm, which makes our algorithm robust against outliers and noise. Finally, we used the EM algorithm to estimate all parameters and obtained closed-form solutions except *γ*, making our registration algorithm computationally efficient.

The data used in preparation of this article are available to the public. The 2D CSFs point sets are segmented from the images from the Retrospective Image Registration Evaluation Project (http://www.insight-journal.org/rire/index.php). The 3D face data can be loaded from http://www.csee.ogi.edu/myron/matlab/cpd/. The 3D lung point sets can be obtained from the Deformable Image Registration Laboratory (http://www.dir-lab.com/index.html.).
